# Automated Counting of Bacterial Colony Forming Units on Agar Plates

**DOI:** 10.1371/journal.pone.0033695

**Published:** 2012-03-20

**Authors:** Silvio D. Brugger, Christian Baumberger, Marcel Jost, Werner Jenni, Urs Brugger, Kathrin Mühlemann

**Affiliations:** 1 Institute for Infectious Diseases, University of Bern, Bern, Switzerland; 2 Bern University of Applied Sciences, Engineering and Information Technology, Burgdorf, Switzerland; 3 Department of Infectious Diseases, University Hospital, Bern, Switzerland; Charité-University Medicine Berlin, Germany

## Abstract

Manual counting of bacterial colony forming units (CFUs) on agar plates is laborious and error-prone. We therefore implemented a colony counting system with a novel segmentation algorithm to discriminate bacterial colonies from blood and other agar plates.

A colony counter hardware was designed and a novel segmentation algorithm was written in MATLAB. In brief, pre-processing with Top-Hat-filtering to obtain a uniform background was followed by the segmentation step, during which the colony images were extracted from the blood agar and individual colonies were separated. A Bayes classifier was then applied to count the final number of bacterial colonies as some of the colonies could still be concatenated to form larger groups.

To assess accuracy and performance of the colony counter, we tested automated colony counting of different agar plates with known CFU numbers of *S. pneumoniae*, *P. aeruginosa* and *M. catarrhalis* and showed excellent performance.

## Introduction

Microbiological research techniques often rely on accurate determination of colony forming units (CFUs). Routinely, this is done by aliquoting a small amount of a liquid culture and plating out several serial dilutions onto culture plates (Petri dishes containing semisolid medium). After incubation in appropriate conditions for the microorganism of choice, the colonies are counted to determine the number of CFU. This is done by manually counting of colonies on plates illuminated by transmitted light. The concentration of bacteria in the original culture can then be calculated based on the assumption that each colony has raised from one single bacterium (colony forming unit, CFU). This process is time-consuming, tedious and error prone. There is a tendency to analyse only high dilutions of the initial culture as these have fewer colonies to count. Unfortunately, in low count assays minor counting errors have significant effects on the calculated concentration in the primary liquid medium.

On the other hand, accurate counting of plates with high numbers of CFUs is error prone since it requires a high level of attention by the performer. Therefore, often only parts of a plate are analyzed and used to estimate the whole plate count after extrapolation [1]. Furthermore high numbers of CFUs on a plate can lead to false redults due to overcrowding of bacteria [2].

This study aimed to design an automated colony counter which reliably detects, bacterial counts and colony size on semisolid agar plates of 85 mm diameter. The system should be suitable for the study of important human pathogens, such as *Streptococcus pneumoniae*, *Moraxella catarrhalis* and *Pseudomonas aeruginosa*. These bacteria are grown on diverse agar plates including Columbia blood sheep agar (CSBA), chocolate agar and brain heart infusion (BHI) agar plates. The system should also be user-friendly and cost-effective with an algorithm that is adaptable to other culture media and microorganisms.

## Methods

### Bacterial strains and growth conditions

A total of 7 clinical isolates of *S. pneumoniae* were selected from two nationwide surveillance programs collecting nasopharyngeal and invasive isolates [3], [4]. Additionally, a clinical isolate of *Pseudomonas aeruginosa* and ATCC strain 25238 of *Moraxella catarrhalis* were used for validation of the automated colony counter (one isolate for each species). For liquid culture all isolates were grown in brain heart infusion (BHI) broth, supplemented with 5% fetal calf serum (FCS) for *S. pneumonia*e. For culture on solid media, *S. pneumoniae and Pseudomonas aeruginosa* were grown on CSBA plates and *Moraxella catarrhalis* on BHI plates. All agar plates were produced in house. Strains were grown at 37°C in a 5% CO_2_ atmosphere. For counting experiments strains were grown in liquid medium to an OD _600 nm_ 0.3–0.4. Ten-fold serial dilutions of culture were made in phosphate buffered saline (PBS, pH 7.4) and 100 µl of dilutions (usually of 10^−4^ to 10^−7^) were plated out on agar plates using glass inoculators and a small rotating disk. Plates were incubated at 37°C overnight in a 5% CO_2_ atmosphere before counting of colonies.

### Colony Counter Hardware Configuration

**Figure 1:**
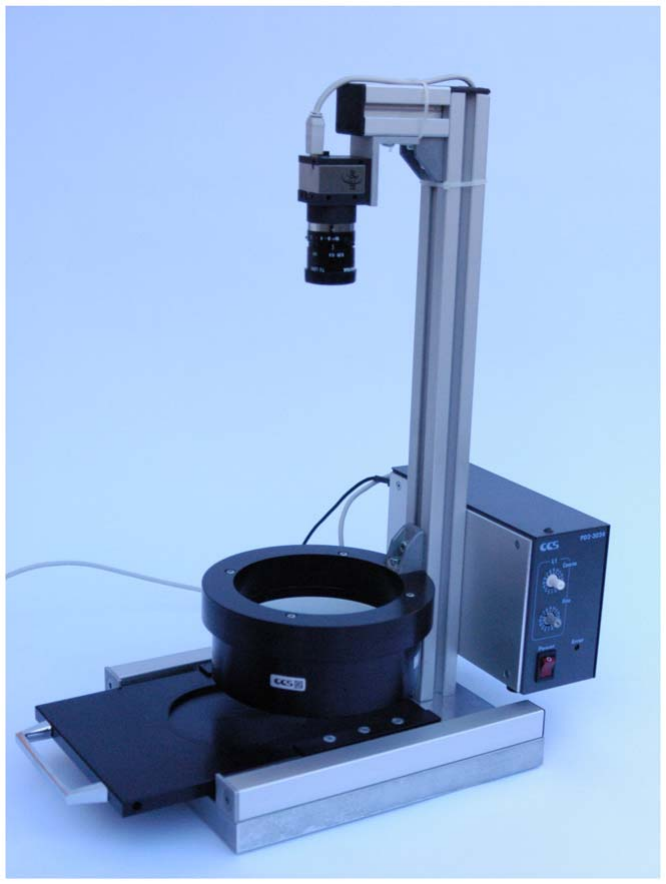
Hardware assembly of the automated colony counter.

The completely installed system is shown in Figure 1. An aluminium rack with a drawer to insert and remove Petri dishes into the machine was constructed. This drawer is eloxadised to minimize light scattering. Attached on the back is a power supply unit combined with a dimmer. This bottom assembly is linked to the camera holding arm with a column. The circular dark field illumination is also attached to the column. Construction plans are added as supplementary information: base plate (Figure S1), drawer (Figure S2) and retaining device for the electronics (Figure S3).

### Colony Counter Illumination and Imaging

Petri dishes were illuminated with a circular dark field illuminator and an IDS uEye UI-1640-C camera with a resolution of 3.3 megapixel (2048×1536 pixel) and a C1614-M lens with 16 mm focal length (both Stemmer Imaging, Pfäffikon, Switzerland). The assembly height of the camera was about 300 mm over the dish. The camera is connected to the computer via a USB interface and the dark field illumination is feeded by the power supply unit.

### Colony Counter Software Algorithms

The software was written in MATLAB 7.9 (The Mathworks, Natick, MA) and a stand-alone application was created, so the software can be used without a complete installation of MATLAB. Indeed, it requires the MATLAB component runtime (MCR) installed on the target computer. Our software was compiled and tested with uEye driver version 3.20.0.2 (www.ids-imaging.de) on Windows XP SP2 (Microsoft, Bern, Switzerland). It is recommended to use this system setup. The application is able to store generated results in xls-files which can be read and edited with MS Excel (Microsoft, Bern, Switzerland).

Software and algorithms are available online (File S1).

### uEye toolbox

This toolbox was created to enable the communication between MATLAB and the uEye driver, as the driver cannot be accessed by MATLAB directly. This toolbox was written in C++. There is a relatively simple way to include C/C++ code into MATLAB scripts. The uEye toolbox provides the functionality to set the colour settings of the camera to RGB24 (red, green and blue and 8 bit resolution per colour channel).

### Counting of bacterial colonies

To assess accuracy and performance of the automated colony counter we compared automated colony counting with routine manual counting and both methods were compared to the gold standard of manual counting performed on high resolution images of plates.

Routine manual counting was performed by 2 independent persons with the help of a transmission light array with magnifier and a handcounter (Tamaco LTD., Taichung, Taiwan). Counted CFU were marked with a pen on the plate cover to discriminate counted from uncounted colonies. Plates with over 200 colonies were usually counted by dividing the plates into equal sectors (from 1/2 up to 1/8). After counting one sector, the count was multiplied with the total number of sectors to estimate whole plate CFU count.

After routine manual counting high resolution images (4.2*10^6^ pixels) of each plate were taken with a FluorChem SP Megapixel Superior Performance Chemiluminescence, Fluorescence and Visible Image System (AlphaInnotec, SanLeandro, CA, USA). Colonies on images displayed on the screen were counted independently by two persons with marking CFUs on the screen as counted.

After routine and high resolution image manual counting automated colony counting was performed once and results were stored in an xls-file generated by the colony counter standalone application.

Automated colony counting and routine manual counting were compared to the gold standard (manual counting on high performance images) by linear regression analysis using Prism 5 software (GraphPad, La Jolla, CA).

## Results

**Figure 2:**
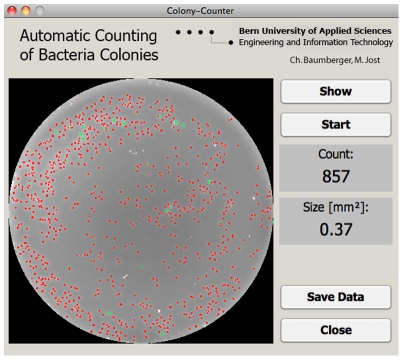
Graphical user interface (GUI) of the automated colony counter. GUI showing a typical result obtained after counting a blood agar plate with pneumococcal colonies. Red: counted as a single bacterial colony. Green: counted as double colonies.

Figure 1 shows complete assembly of the colony counter and Figure 2 the graphical user interface.

### Illumination

**Figure 3:**
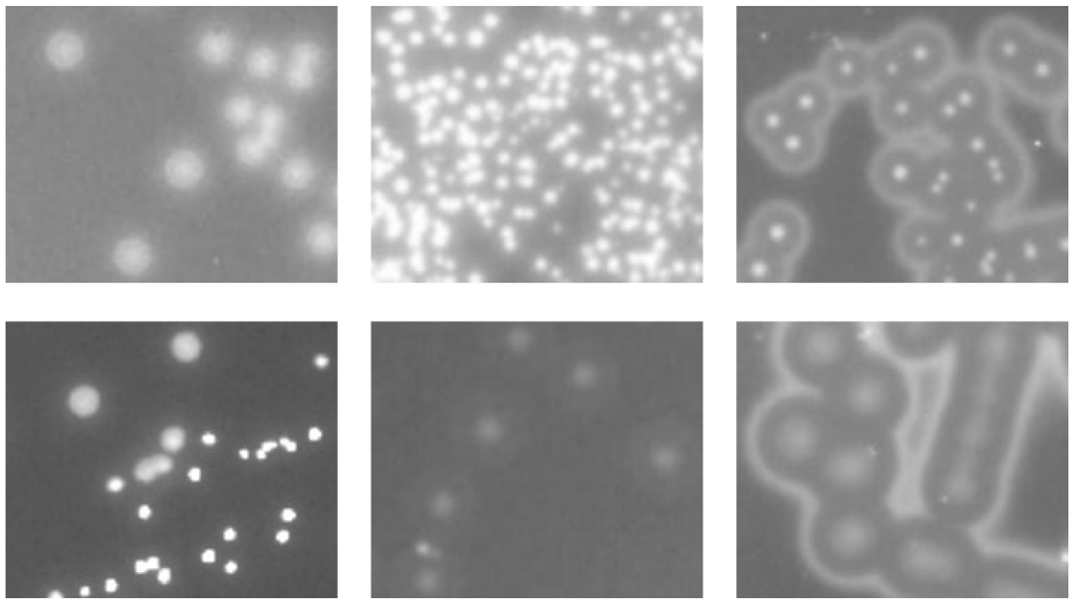
Colony morphologies. Blue channel of RGB images showing different colony morphologies of three different *S. pneumoniae* strains grown on blood agar plates.

A key component of our colony counting system is the choice of illumination. Different illumination techniques like front and back lighting were tested. Since the surface of the blood agar reflects most of the light, images where illuminated with a direct front light were heavily deranged. Transmitted lighting illumination gave good object background discrimination but due to the inhomogeneity of the agar thickness the discrimination was only possible for a limited area of the plate. As bacterial colonies are usually slightly elevated from the agar surface dark field illumination was evaluated. In contrast to front lighting illumination, the light beam of the dark field illuminator is projected from the side onto the target object. With white light dark field illumination the background of the blood agar became prominent, which impaired colony discrimination, but this problem was solved by using a blue dark field light source. Blue dark field illumination gave the best discrimination of colonies and medium background especially in the blue part of collected RGB images (Figure 3). Therefore only the blue part of the image was used for further image processing. A disadvantage of dark field illumination is the influence of dust on the medium. Such undesirable distracters had to be removed from the green colour channel in the image purification steps (see below).

### Colony-segmentation algorithm

**Figure 4:**
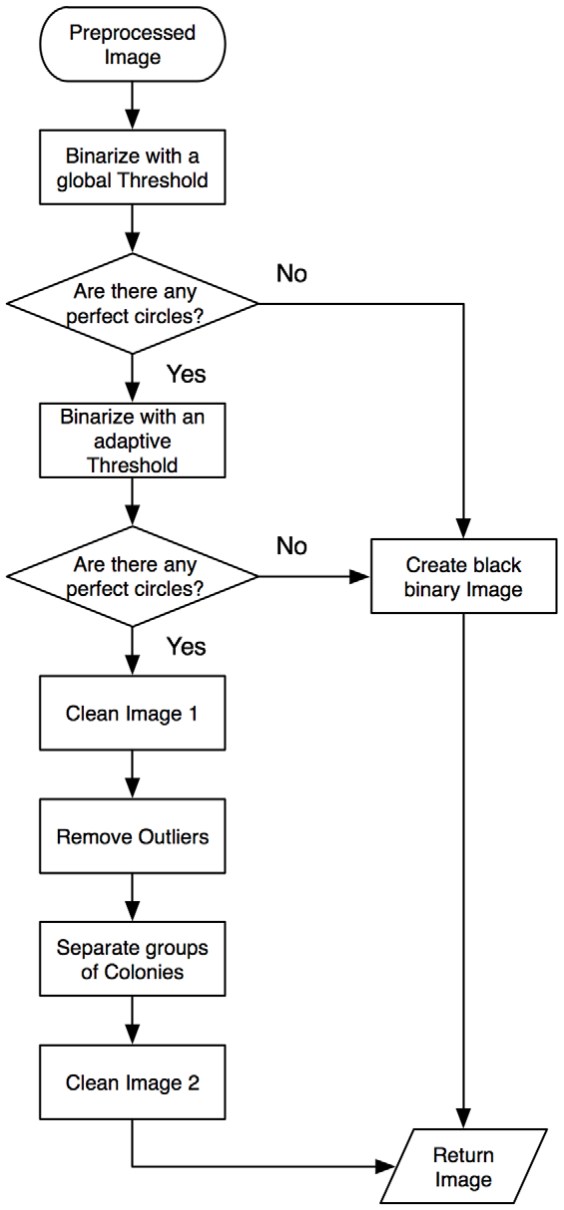
Flowchart of the colony separation and counting algorithm.

A flowchart describing the segmentation algorithm is shown in Figure 4. After an image of an agar plate has been taken, it has to be pre-processed with Top-Hat-filtering [5] in order to obtain a uniform background by removing inhomogeneity of the semisolid agar layer. This is followed by the segmentation step, during which the colony images are extracted from the blood agar and individual colonies are separated. A Bayes classifier is then applied to count the final number of bacterial colonies. This step is necessary as some of the colonies are still concatenated to form larger groups. A Bayes classifier is a simple probabilistic classifier based on applying Bayes theorem. Geometric properties such as ratio between major and minor axis of the group are used to verify the number of colonies contained in the group. In subsequent sections, the novel segmentation algorithm is described in more detail.

### First binary conversion

First, the pre-processed image is binarized using a dynamic, global threshold, calculated based on the method of Otsu [6]. Binarizing can be understood to set all the pixels with a greyscale value greater than a threshold to one and zero otherwise.

### Search for perfect circles

Perfect circles are searched based on the binary image by analyzing the ratio of major to minor axis length (circle has a ratio of one) and the ratio of object area to the smallest possible surrounding rectangle, the so-called bounding box: 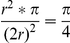 of each found object. Both used properties are well known in the pattern recognition literature and commonly used [7], [8]. When a real world image is discretized to a pixel matrix, no object matches the perfect circle criterion. Therefore, “perfect” circles have to lie within a tolerance of ±20 percent. If by this definition no circles are found, a black binary image is the output.

### Second binarization by adaptive thresholding

Before performing a second binarization, the area outside the Petri dish is removed and pixel values are set to zero. Since the abrupt change from black area to the red blood agar causes a thick rim after binarization, the pixel values are changed to the value of the mean chrominance of the outer 10 pixels within the agar. The background of the image is set to black (adjacent corners of a square image on round plate).

One of the challenges comes from the fact that bacterial strains from the same species may exhibit different colony phenotypes (as shown in Figure 3), which makes it impossible to define a common threshold for colony size. One way to solve this problem is to apply an adaptive threshold where a threshold for each pixel is calculated based on its neighbouring pixels [9]. The assumption behind this method is that smaller image regions are more likely to have approximately uniform illumination, thus being more suitable for setting a threshold. The used method statistically examines the mean intensity values of the local neighbourhood of each pixel. To avoid noise and ensure that a big homogeneous area is segmented as a continuous object, a global threshold is subtracted from the local threshold. The target pixel 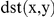 is defined as 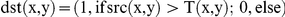 where 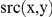 is the grey scale value of the input image pixel and 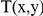 is a local threshold, calculated individually for each pixel and defined as follows: 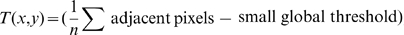, where n is the number of neighbouring pixels.

### First image purification

As a consequence of the binarization process, large connected areas arise on the border of the blood agar. The colonies that touch these boundary parts and the areas are removed.

### Remove outliers

Errors, such as scratches, dust or air bubbles in the agar are best visible in the green channel of the colour image. Based on the mean brightness of the perfect circles a filter for the image's green part is estimated and error objects are removed.

### Separate groups of colonies

The adaptive binarization process results in concatenating several colonies to groups of to four or five individual colonies (confluent colonies on plates). The algorithm performs a distance transformation on the binarized image and segmentation is then done with a watershed transformation [10], [11].

### Second image purification

The application of morphological opening rounds all the sharp corners produced by the watershed transformation [11]. Furthermore, objects smaller than a certain threshold and larger than a second threshold are removed. Both thresholds are computed from the size of the perfect circles.

After the segmentation algorithm is completed, the Bayes classifier distinguishes the remaining concatenated groups into classes of one, two, three or four containing colonies and the final colony counting is proceeded. One important geometric property of different classes is the abrupt change of the angle around an object boundary. If a group contains for example two colonies, then two such abrupt changes can be observed at the borders where the colonies touch each other. Visual inspection showed that the segmentation algorithm discriminated almost all individual bacteria colonies from the agar and separated most of the concatenated groups.

### Performance of colony counting algorithm

**Figure 5:**
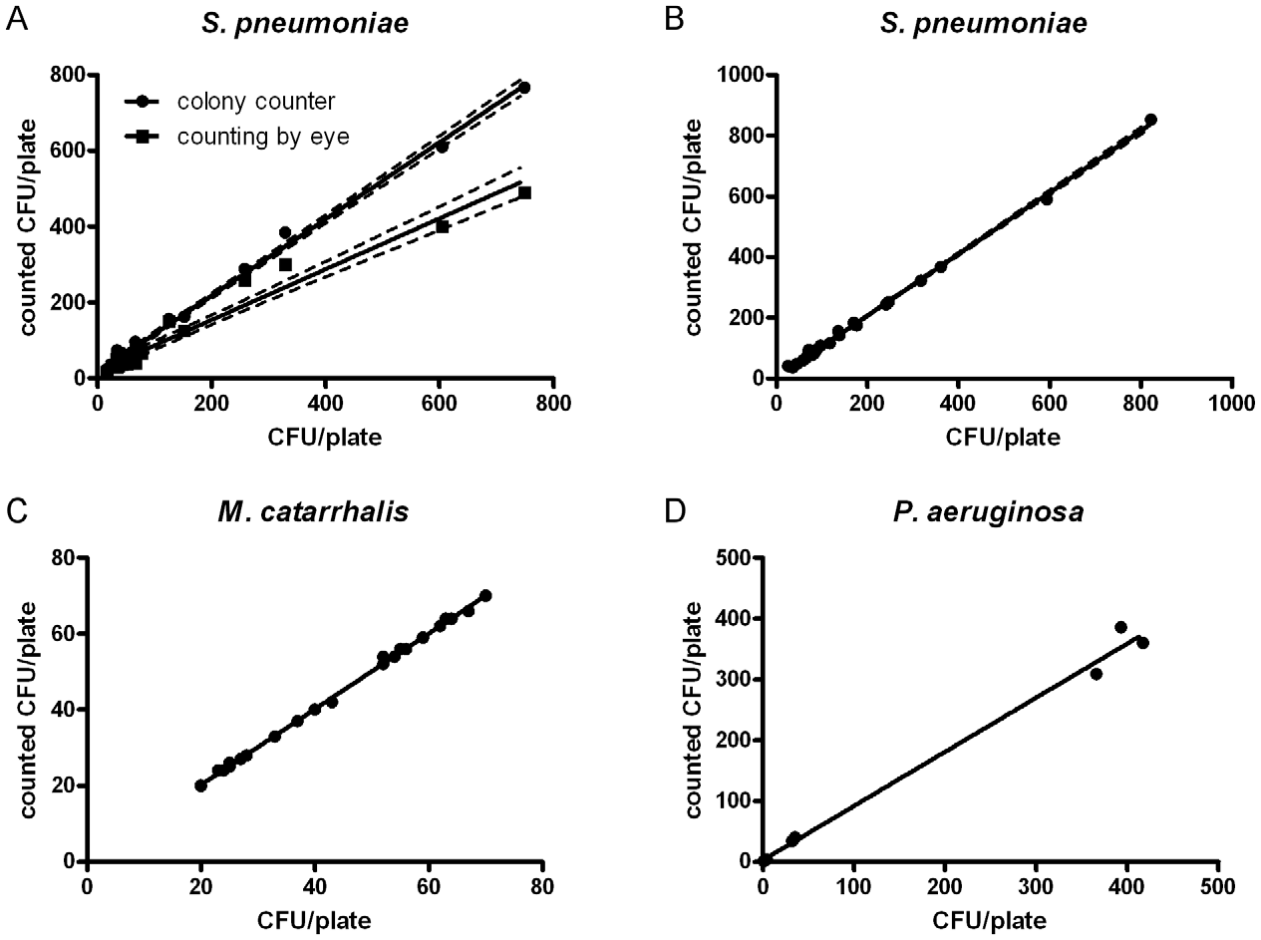
Performance of the automated colony counter. **A.** Performance of automated colony counting (dots) of *S. pneumoniae* CFUs and routine manual counting (squares) versus the gold standard of manual counting on high performance images (X-axis). A total of 22 plates of 7 different pneumococcal strains were analyzed (CFU range 16–749; mean 133.5, median 55.5). Linear regression analysis of manual and automated colony count versus the gold standard showed a significant difference of the two slopes (p<0.0001) with a slope of 1.01(SD ±0.016; 95% confidence interval (CI) 0.98–1.04) for the automated colony counter and a slope of 0.67(SD±0.03; CI 0.61–0.73) for routine manual counting (95% confidence interval is indicated with dotted lines). **B.** Performance of automated colony counting (dots) of *S. pneumoniae* CFUs on blood agar plates versus the gold standard of manual counting on high performance images (X-axis). A total of 26 plates of 7 different pneumococcal strains (CFU range 36–853; mean 176, median 101.5) were analyzed. Linear regression showed a slope of 1.02 (SD±0.01; CI 1–1.03) (95% confidence interval is indicated with dotted lines). **C.** Performance of automated colony counting (dots) of *M. catarrhalis* CFUs on BHI plates versus the gold standard of manual counting on high performance images (X-axis). 25 plates of *M. catarrhalis* strain 25238 were used (CFU range 20–70, mean 43.9, median 42). Linear regression analysis of automated colony count versus the gold standard showed a slope of 1 (SD ±0.008; CI 0.981–1.033) for the automated colony counter. **D.** Performance of automated colony counting (dots) of *P. aeruginosa* CFUs on blood agar plates versus the gold standard of manual counting on high performance images (X-axis). 8 plates of *P. aeruginos*a clinical isolate (strain 460229) were analyzed (CFU range 1–386, mean 142.3, median 37). Linear regression analysis of automated colony count versus the gold standard showed a slope of 0.89 (SD ±0.033; CI 0.81–0.97) for the automated colony counter.

Counting of *S. pneumoniae* colonies was done with all three methods for a total of 7 pneumococcal strains grown on a total of 22 plates with CFU counts ranging from 16 to 749 (mean 133.5 colonies, median 55.5 colonies). Linear regression analysis of manual and automated colony count versus the gold standard showed a significant difference of the two slopes (Figure 5A, p<0.0001) with a slope of 1.01(SD ±0.016; 95% confidence interval (CI) 0.98–1.04) for the automated colony counter and a slope of 0.67(SD±0.03; CI 0.61–0.73) for routine manual counting.

In a second independent experiment using 7 strains grown on 26 plates with CFU counts ranging from 36 to 853 (mean 176, median 101.5) automated colony counting was compared with the gold standard only. Linear regression showed a slope of 1.02 (SD±0.01; CI 1–1.03) (Figure 5B).

Counting of *M. catarrhalis* was done with ATCC isolate (strain 25238) comparing automated colony counting with the gold standard of manual counting performed on high resolution images of plates. 25 plates with CFU counts ranging from 20 to 70 were used (mean 43.9, median 42). Linear regression analysis of automated colony count versus the gold standard of 26 plates showed a slope of 1 (SD ±0.008; CI 0.981–1.003) for the automated colony counter (Figure 5C).

Counting of *P. aeruginosa* was done with a clinical isolate (strain 460229) comparing automated colony counting with the gold standard of manual counting performed on high resolution images of plates. 8 plates with CFU counts ranging from 1 to 386 were used (mean 142.3, median 37). Linear regression analysis of automated colony count versus the gold standard showed a slope of 0.89 (SD ±0.033; CI 0.81–0.97) for the automated colony counter (Figure 5D).

## Discussion

In this study, we present an automated system for accurate counting of bacterial colonies of the human pathogens *S. pneumonia*, *M. catarrhalis* and *P. aeruginosa*. The system works well on different solid growth media including those with a dark background colour such as sheep blood agar. Performance was superior to routine manually counting of plates especially in the presence of higher numbers of colonies. Comparison with the gold standard of counting single colonies on high resolution images showed excellent correlation.

Several automated colony counting systems are commercially available such as the ProtoCOL automated counters or the Whitley aCOLyte (Synbiosis, Cambridge, UK) and the AID BacSpot (AID, Strassberg, Germany) [12], [13]. These systems have been designed for quality control in food production industry and can therefore handle large number of samples with many different bacterial species. They are not widely used in research laboratories due to their relative high price. Also, to our knowledge, there has been no evaluation of commercially available systems for counting bacterial strains such as *S. pneumoniae* grown on blood agar plates.

The system presented in this study is a low priced alternative to the high-throughput systems with an open source software code. Therefore, the software can be adapted by users to their individual needs (other organisms, other growth media, etc.). The hardware is easy to assemble and total price is less than 8'000 USD.

The performance of applications for automated bacterial colony counting has been reported before. Whereas some authors describe the performance of commercial counters [12], others describe novel algorithms for colony counting and their performance [13], [14], [15]). Cordiki and colleagues presented a complete colony counting system and showed excellent performance with yeast and different non-pneumococcal bacterial species grown on (not further specified agar plates with a bright background colour. An epi-illumination system with a light source at an angle between 30–40° from the vertical axes was used. In our study we used a ring shaped homogenous light source to ensure equal illumination. Cordiki et al. used commercial image analysis software for image processing and the standard software was improved by integrating a multilevel threshold algorithm showing a regression equation of y = 0.9753 for tested species [15]. However, the authors did not test this system with *S. pneumoniae* and not with bacteria grown on blood agar plates.

Putman and colleagues presented a counting method using the ProtoCol software [13]. But this method required staining of colonies with dyes such as 2,3,5-triphenyl tetrazolium chloride (TTC) to increase the contrast between bacterial colonies [13], [16]. They showed that the ProtoCOL counter counts were about 10–15% lower than true counts and this deviation was noticeable when the numbers of colonies exceeded fifty. The authors were able to improve the performance of the ProtoCOL by supplying images generated by scanning Petri dishes with a common document scanner. Since source codes for the commercial systems are not available a comparison of our system to those algorithms was not possible.

An advantage of the automated colony counter system described here is that CFUs can be counted without prior staining. After counting, colonies can therefore be used for further experiments. Despite the excellent performance, a remaining difficulty is the recognition of dust or scratches especially on blood agar plates where they exhibit high diversity of shapes and appearances.

In summary, the main contribution of this work is a novel segmentation algorithm, which allows for cutting the background from the foreground (i.e. from the colonies) and is robust to colony size as well as to different textures and appearances of the colonies. Included in this algorithm is an efficient way to separate connected groups of colonies. Performance of this system was shown to be equal the gold standard of hand counting high resolution images but in much less time.

## Supporting Information

Figure S1 **Construction plan for the colony counter ground plate.**
(PDF)

Figure S2 **Construction plan for the colony counter drawer.**
(PDF)

Figure S3 **Construction plan for the colony counter retaining device.**
(PDF)

File S1 **Colony counter software files: stand-alone application (cc.exe) and supporting files.** Please note that the MATLAB component runtime (MCR) has to be installed on the target computer to use the software. MCR can be downloaded from the mathworks website (http://www.mathworks.com).
(RAR)
