# Supplementary figures and images for: Automated Counting of Bacterial Colony Forming Units on Agar Plates
Source: PLoS One. 2012 Mar 20;7(3):e33695. doi: 10.1371/journal.pone.0033695 (PMC3308999; doi:10.1371/journal.pone.0033695)

22.11.2011 Hecsa

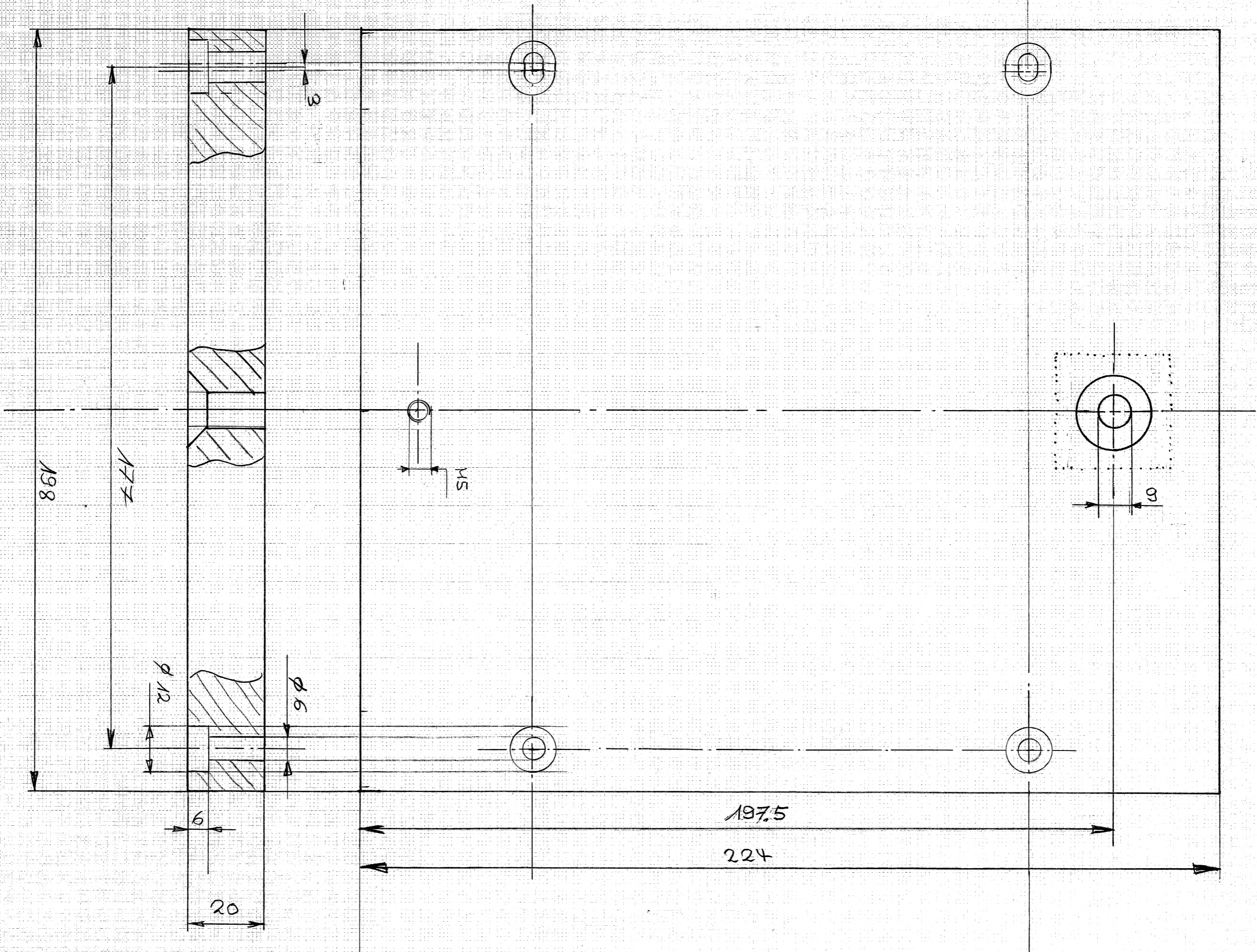

Grundplatte  
A1

Pos. 01

Supplement: Figure S1 — Construction plan for the colony counter ground plate. (PDF) [file pone.0033695.s001.pdf]

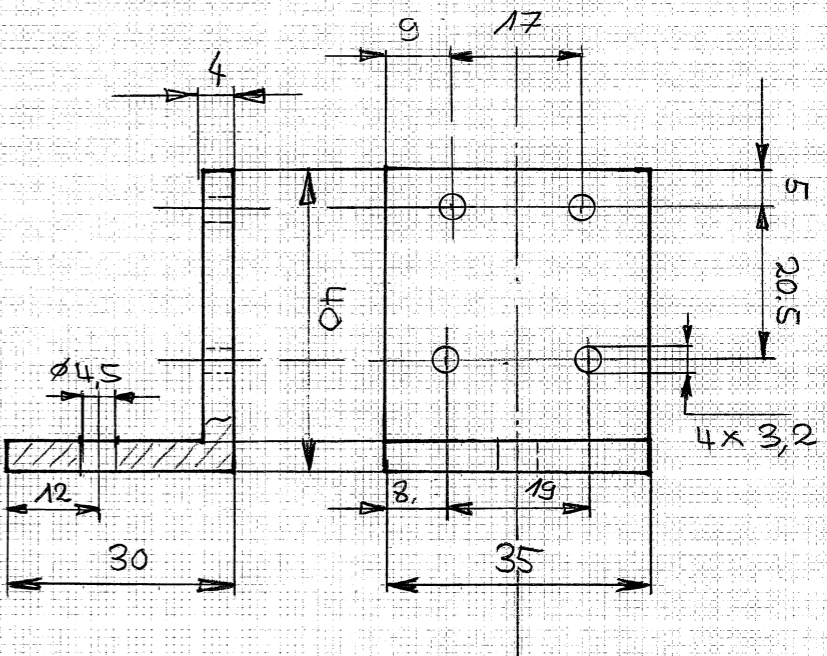

WINKEL KAMERA

Aluminium

Pos. 09

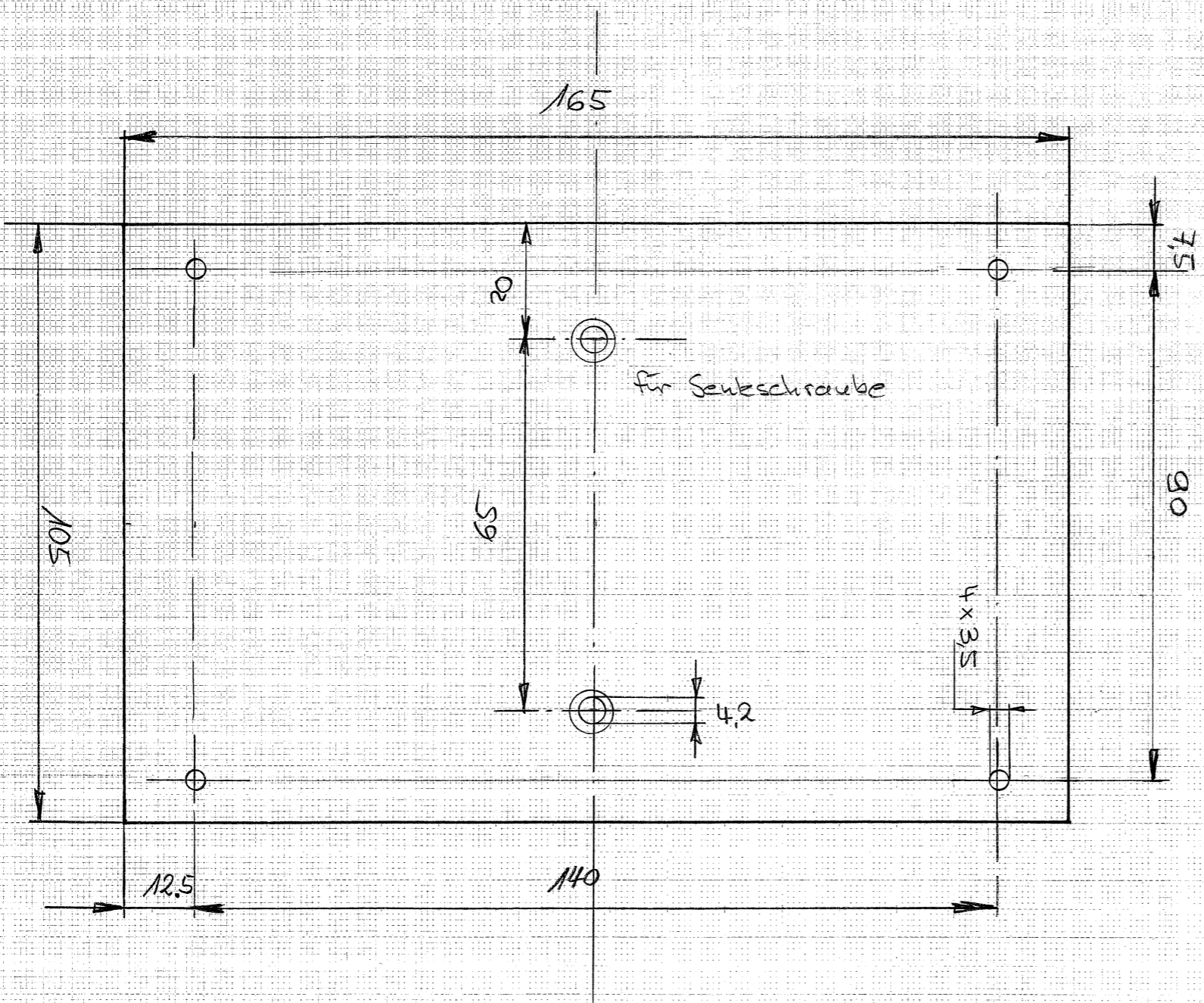

HALTERPLATTE ELEKTRONIK

Aluminium dicke 2mm

Pos. 08

23.11.2011 HGW

Supplement: Figure S3 — Construction plan for the colony counter retaining device. (PDF) [file pone.0033695.s003.pdf]
