# Supplementary material for: Automated Counting of Bacterial Colony Forming Units on Agar Plates
Source: PLoS One. 2012 Mar 20;7(3):e33695. doi: 10.1371/journal.pone.0033695 (PMC3308999; doi:10.1371/journal.pone.0033695)
Supplement: Figure S2 — Construction plan for the colony counter drawer. (PDF) [file pone.0033695.s002.pdf]

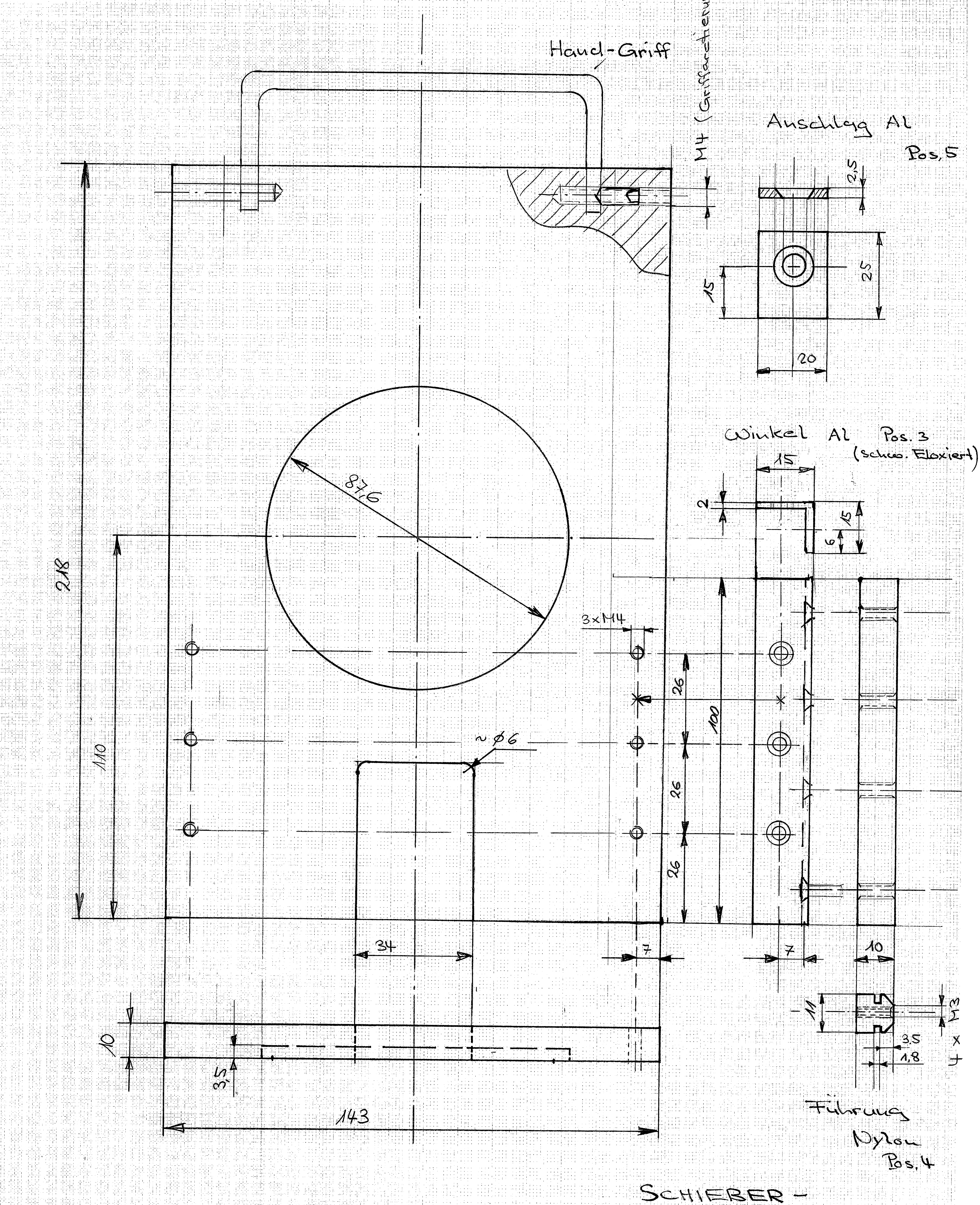

SCHIEBER -
GRUNDPLATTE
(schwarz Eloxiert)
Aluminium 218 × 143 / 10
Pos. 02.

22.11.2011 HGW
